# Resveratrol Regulates Colorectal Cancer Cell Invasion by Modulation of Focal Adhesion Molecules

**DOI:** 10.3390/nu9101073

**Published:** 2017-09-27

**Authors:** Constanze Buhrmann, Parviz Shayan, Ajay Goel, Mehdi Shakibaei

**Affiliations:** 1 Musculoskeletal Research Group and Tumour Biology, Chair of Vegetative Anatomy, Institute of Anatomy, Faculty of Medicine, LMU Munich, Pettenkoferstrasse 11, D-80336 Munich, Germany; constanze.buhrmann@med.uni-muenchen.de; 2 Department of Parasitology, Faculty of Veterinary Medicine, University of Tehran, Tehran 141556453, Iran; pshayan@ut.ac.ir; 3 Investigating Institute of Molecular Biological System Transfer, Tehran 1417863171, Iran; 4 Center for Gastrointestinal Research, Center for Translational Genomics and Oncology, Baylor Scott & White Research Institute and Charles A Sammons Cancer Center, Baylor University Medical Center, Dallas, TX 75246, USA; Ajay.Goel@BSWHealth.org

**Keywords:** resveratrol, colorectal cancer, FAK, NF-κB, integrin, Sirt1

## Abstract

Resveratrol, a safe and multi-targeted agent, has been associated with suppression of survival, proliferation and metastasis of cancer, however, the underlying mechanisms for its anti-cancer activity, particularly on cellular signaling during cancer cell migration still remain poorly understood. We investigated the invasion response of two human colorectal cancer (CRC) cells (HCT116 and SW480) to resveratrol and studied the effect of specific pharmacological inhibitors, cytochalasin D (CytD) and focal adhesion kinase-inhibitor (FAK-I) on FAK, cell viability and migration in CRC. We found that resveratrol altered cell phenotype of both CRC cells, reduced cell viability and the results were comparable to FAK-I and CytD. These effects of resveratrol were associated with marked Sirt1 up-regulation, FAK down-regulation, inhibition of focal adhesion and potentiation of effects by combinatorial treatment of resveratrol and inhibitors. Interestingly, inhibition of FAK with FAK-I or treatment with CytD suppressed resveratrol-induced Sirt1 up-regulation and markedly down-regulated FAK expression. Resveratrol or combination treatment with inhibitors significantly activated caspase-3 and potentiated apoptosis. Moreover, resveratrol suppressed invasion and colony forming capacity, cell proliferation, β1-Integrin expression and activation of FAK of cells in alginate tumor microenvironment, similar to FAK-I or CytD. Finally, we demonstrated that resveratrol, FAK-I or CytD inhibited activation of NF-κB, suppressed NF-κB-dependent gene end-products involved in invasion, metastasis, and apoptosis; and these effects of resveratrol were potentiated by combination treatment with FAK-I or CytD. Our data illustrated that the anti-invasion effect of resveratrol by inhibition of FAK activity has a potential beneficial role in disease prevention and therapeutic management of CRC.

## 1. Introduction

As the third most prevalent cancer in the world, colorectal cancer affects more than half a million people per annum [1] constituting about 10% of all estimated cancer deaths [2]. Although a long-term decline in colorectal cancer death has been observed since the early 1980s, mainly related to enhanced screening and improved treatment methods, colorectal cancer remains a clinical challenge as ~50% of patients develop chemoresistance to current chemotherapeutic drugs, as well as recurrence and metastatic events [3,4]. The pathogenesis of colorectal cancer is regarded as a multi-step process, orchestrated by multiple genetic mutations, epigenetic modifications and interaction with the tumor microenvironment [5]. It is recognized that the interaction between the malignant tumor cells and their surrounding microenvironment are important mediators that trigger tumor progression, invasion and metastasis [6,7].

The Focal Adhesion Kinase (FAK) is a non-receptor tyrosine kinase that is activated in response to cell adhesion and transduces extracellular signals through tyrosine phosphorylation onto various intracellular molecules in both growth factor dependent and adhesion-dependent manner [8]. FAK is expressed in most tissues and cell types and is evolutionarily conserved in mammalian species as well as lower eukaryotic organisms [9]. Indeed, the human FAK protein is structurally identical to both mouse and chicken FAK and expresses 95–97% amino acid homology between these species, suggesting a crucial function in cells [10].

FAK participates in integrin-mediated signaling functions, enabling the cell adhesion signals that stimulate reorganization of the cytoskeleton and interaction of the cells with their microenvironment [11,12]. Integrins are cell surface receptors mediating e cell adhesion to the extracellular matrix, as well as dynamic signaling molecules facilitating cell migration as it occurs in normal processes such as angiogenesis, wound healing, immune system function, and development [13]. Indeed, integrin-mediated signaling is one of the strongest activators of FAK [14]. Integrin receptor binding to extracellular matrix proteins results in the formation of adhesomes where FAK localizes to a membrane-proximal signaling layer containing integrin cytoplasmic tails and paxillin and FAK can function upstream of Src phosphorylation events [14].

It is well known that integrins act as bidirectional signaling transmitters linking the signaling communication between the inside and the outside of the cells [15]. Aberrant expression and function of integrins may contribute to many disease states including cancer [13]. In cancer, it has been observed that integrin-mediated signaling via focal adhesion enables the reorganization of the cytoskeleton and favors tumor progression, invasion and metastasis [15]. Aggregation of FAK with integrins and cytoskeletal proteins in focal contacts leads to increased cell migration, as well as potential regulation of cell proliferation and survival [16,17]. Additionally, in cancer, FAK has been shown to be involved in cellular movement, invasion, survival, gene expression and cancer stem cell self-renewal [18]. Genetic tumor profiling has revealed both FAK DNA amplification (*PTK2* gene at 8q24.3) and elevated FAK mRNA levels in several cancers, including breast and ovarian carcinomas [19]. Indeed, activation of FAK has been shown to be high in metastatic aggressive tumors and is correlated with poor clinical outcome [8].

The plant-derived polyphenol, resveratrol (3,5,4′-trihydroxy-trans-stilbene), is found in more than 70 common plant species, including red grapes, cranberries, peanuts and root extracts of the weed *Polygonum cuspidatum* [20,21,22]. Several reports have suggested that resveratrol modulates multiple cellular signaling pathways through diverse mechanisms and thus is a promising multi-targeted agent that can suppress cancer cell proliferation, metastasis, and induce apoptosis [23,24,25,26]. Moreover, it has been previously reported that resveratrol inhibits IκB-kinase-β-mediated NF-κB activation and it is a potent natural activator of Sirtuin-1 (Sirt1)—a nucleus related NAD+ histone deacetylase class III [27,28,29]. Interestingly, previous reports from our laboratory have shown that resveratrol exerts its inhibitory effects in colorectal cancer through its activity on diverse subcellular targets, including NF-κB and Sirt1 and inhibition of epithelial-to-mesenchymal transition (EMT) markers with upregulation of intercellular junctions and E-cadherin and the downregulation of NF-κB and vimentin [26,30]. Interestingly, the inhibition of EMT by resveratrol has been associated with modulation of integrin activity [31]. Additionally, resveratrol has been shown to decrease the levels of cell adhesion proteins and EMT associated mediator α5β1 integrin and hyaluronic acid in ovarian cancer cell lines [32]. Further, it was recently shown that resveratrol is able to inhibit phosphorylation of FAK in several cell lines including the colon cancer cell line HT-29 [33,34,35].

In view of the above-mentioned findings, in the present study, we investigated the effect of resveratrol on the regulation of colorectal cancer cell invasion and metastasis through modulation of focal adhesion molecules and cancer cell motility.

## 2. Materials and Methods

### 2.1. Antibodies

Monoclonal anti-phospho-specific-FAK and anti-FAK antibodies were obtained from Becton Dickinson (Heidelberg, Germany). Anti-Sirt1 and anti-CXCR4 (CXC-Motiv-Chemokinreceptor 4) antibodies were purchased from Abcam PLC (Cambridge, UK). Anti-phospho-specific p65 (NF-κB) and anti-phospho-specific p50 (NF-κB) antibodies were obtained from Cell Technology (Beverly, MA, USA). Anti-active caspase 3, anti-MMP-9 and anti-MMP-13 antibodies were obtained from R&D Systems (Heidelberg, Germany). Monoclonal anti-β1-Integrin and anti-β-actin antibodies were purchased from Sigma-Aldrich Chemie (Munich, Germany). Monoclonal Anti-β-Actin antibody was obtained from Santa Cruz Biotechnology (Santa Cruz, CA, USA). Alkaline phosphatase–linked sheep anti-mouse and sheep anti-rabbit secondary antibodies for immunoblotting were purchased from EMD Millipore (Schwalbach, Germany). Anti-Ki-67 and secondary antibodies used for fluorescence labeling were obtained from Dianova (Hamburg, Germany). All antibodies were used at concentrations recommended by the manufacturers.

### 2.2. Growth Media and Chemicals

Cell culture growth medium consisting of Dulbecco’s modified Eagle’s medium/Ham’s F-12 (1:1), 10% fetal bovine serum (FBS), 0.5% amphotericin B solution, 1% penicillin/streptomycin solution (10,000 IU/10,000 IU), 75 μg/mL ascorbic acid, 1% essential amino acids and 1% glutamine was obtained from Seromed (Munich, Germany). Epon was purchased from Plano (Marburg, Germany). Alginate, cytochalasin D (CytD) and resveratrol with purity greater than 98% were purchased from Sigma. A 100 mM stock solution of resveratrol (molecular weight 228.2) was prepared in ethanol and further diluted in cell culture medium to prepare working concentrations. The maximum final content of ethanol in cultures was less than 0.1% and this concentration was also used as a control. CytD was dissolved in DMSO and further diluted in serum-starved medium to establish working solutions. Hereby, final concentrations of DMSO did not exceed 0.1%. Focal adhesion kinase inhibitor (PF-562271 and PF-573228) was purchased from Sellekchem (Munich, Germany). For the experiments, a stock solution of 10 mM Focal adhesion kinase inhibitor (FAK-I) dissolved in DMSO was prepared and further diluted in serum-starved medium to establish working solutions. All stock solutions were stored as recommended by the manufacturers.

### 2.3. Cell Lines and Cell Culture

Human SW480 colorectal cancer (CRC) cells were purchased from the American Type Culture Collection (ATCC, Manassas, VA, USA) and human HCT116 CRC cells were obtained from the European Collection of Cell Cultures (Salisbury, UK). The cells were maintained in tissue culture flasks in growth medium and in a humidified incubator at 37 °C in an atmosphere of 95% air and 5% CO_2_. The medium was changed every three days, and cells were passaged at 80–90% confluency using trypsin/EDTA.

### 2.4. Immunofluorescence and Light Microscopy Analysis of Monolayer Cultures

To examine the influence of FAK-inhibitor, CytD and resveratrol on HCT116 and SW480 CRC cell phenotype and expression/activation of Sirt1, FAK, Ki-67, and caspase-3, light microscopic investigations and immunofluorescence staining were performed. Cells were cultured on glass plates to sub-confluency in monolayers and either left untreated, or were treated with 5 µM resveratrol, with 10 µM FAK-I (PF-562271), with 0.1 µg/mL CytD or a combination of 5 µM resveratrol and either 10 µM FAK-I (PF-562271) or 0.1 µg/mL CytD for 12 h. For immunofluorescent investigation, cells were fixed with methanol, rinsed with PBS and incubated with bovine serum albumin (BSA) for 30 min. Primary antibodies were diluted 1:50 in PBS/BSA, incubated overnight at 4 °C in a humidfied chamber, washed three times with PBS/BSA followed by incubation with rhodamine-coupled secondary antibodies for 1.5 h and finally washed again three times with aqua dest. Counter staining was performed with DAPI (4,6-diamidino-2-phenylindole, Sigma) to visualize cell nuclei. Slides were covered with fluoromount and examined under a fluorescent microscope (Leica, Darmstadt, Germany). In FAK immunolabeled cultures the number of focal adhesion points was quantified by scoring 800–1000 cells from 10 different microscopic fields. One of three independent experiments is shown. Values were compared to the control, and statistically significant values with *p* < 0.05 are designated by an asterisk (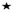); *p* < 0.01 by two asterisks (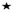

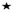
).

### 2.5. DAPI Staining of Apoptotic Cells

To examine the apoptotic changes induced by two different FAK inhibitors (PF-562271 and PF-573228) in HCT116 and SW480 CRC cells, DAPI (4,6-diamidino-2-phenylindole, Sigma) nuclear staining assay was performed. Cells were seeded on glass plates at 80–90% confluency and treated with different concentrations of FAK-I (PF-562271: 0.1, 1, 10, 20, and 50 µM; and PF-573228: 0.1, 1, 10, 20, and 50 µM) for 12 h. After completion of treatment, the cells were fixed with methanol for 10 min, washed twice with PBS, and incubated with DAPI solution for 10 min in the dark. Labeled cells were washed repeatedly with PBS to remove the excess DAPI stain and evaluated under fluorescence microscope (Leica, Darmstadt, Germany). Quantification of apoptotic cells was performed by scoring 800–1000 cells from 10 different microscopic fields. Result from one of three independent experiments is shown in figures. Values were compared to the control, and statistically significant values with *p* < 0.05 are designated by an asterisk (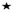); *p* < 0.01 by two asterisks (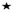

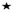
).

### 2.6. Cell Proliferation Assay

The effect of FAK-inhibitor, CytD, resveratrol and their combination on the proliferation and viability of HCT116 and SW480 CRC cells was determined by the 3-(4,5-dimethylthiazol-2-yl)-2,5-diphenyltetrazolium bromide (MTT) uptake method as described previously [36]. Briefly, the cells (5000 per well in triplicate in a 96-well plate) were treated with either 5 µM resveratrol alone, different concentrations of CytD (0.1, 1, 2, and 4 µg/mL), different concentrations of FAK-I (PF-562271: 0.01, 0.1, 1, and 10 µM) each or co-treated with 5 µM resveratrol and the indicated concentrations of either CytD or FAK-I for 12 h. Subsequently, 100 µL of modified cell culture medium (DMEM without phenol red, without ascorbic acid and only 3% FBS) and 10 µL MTT solution (5 mg/mL) was added to each well and the plate was incubated for 4 h at 37 °C. Finally, 100 µL of the MTT solubilization solution (10% Triton X-100/acidic isopropanol) was added per well, and the cells incubated overnight at 37 °C. Metabolically active cells were evaluated by measuring the Optical Density at 550 nm (OD_550_) using a 96-well multi-scanner plate ELISA reader (Bio-Rad Laboratories Inc. Munich, Germany). Data were derived from at least three independent experiments, and statistical analysis was done to obtain the final values.

### 2.7. Alginate Culture

A detailed description of cell cultivation in alginate culture has been previously described by our group [26,30,37]. Briefly, HCT116 and SW480 CRC cells (1 × 10^6^/mL) were resuspended in alginate (2% in 0.15 M NaCl) and added dropwise into a 100 mM CaCl_2_ solution at ambient temperature (AT) where the alginate beads polymerized after 10 min forming a round and stable bead. Subsequently, the alginate beads were washed three times with 0.15 M NaCl solution and twice with serum-starved medium (3% FBS) before starting treatment.

### 2.8. Invasion (Migration) Assay

To investigate invasion capacity, HCT116 and SW480 CRC cells were cultured in alginate beads as described above. Cells were either left untreated, or treated with 5 µM resveratrol alone, different concentrations of CytD (0.1, 1, and 2/mL), FAK-I (PF-562271: 0.01, 0.1, 1, and 10 µM) individually or co-treated with 5 µM resveratrol and the indicated concentrations of either CytD or FAK-I for 28 days. Invasive cells that migrated through the alginate beads and formed adhered colonies on the bottom of the petri dish were fixed with Karnowsky fixative, stained with toluidine blue for 5 min and carefully washed two times with PBS. The number of migrated and positively stained adhered colonies were quantified and evaluated manually by counting all colonies under a light microscope (Axioplan, Zeiss, Germany) and visualized. This assay was repeated every 3 to 4 days until Day 28 of culture. The mean number of colonies in triplicate was calculated, which is reported in each graph. Each experiment was repeated at least three times.

### 2.9. Western Blot Analysis

In another set of experiments, HCT116 and SW480 CRC cells were cultured in alginate beads as described above and were either left untreated, or treated with 5 µM resveratrol alone, 0.1 µg/mL CytD, 10 µM FAK-I (PF-562271) or co-treated with 5 µM resveratrol and either the indicated concentration of CytD or FAK-I for 10 days. Proteins were extracted from the alginate cultures with lysis buffer (50 mM Tris-HCl, pH 7.2, 150 mM NaCl, 1% (*v*/*v*) Triton X-100, 1 mM sodium orthovanadate, 50 mM sodium pyrophosphate, 100 mM sodium fluoride, 0.01% (*v*/*v*) aprotinin, 4 µg/mL of pepstatin A, 10 µg/mL of leupeptin, 1 mM phenylmethylsulfonyl fluoride, PMSF) on ice for 30 min, as previously described [38] and total protein concentration was measured with the bicinchonic acid assay system (Uptima, Monlucon, France) using bovine serum albumin as a standard. Subsequently, samples were reduced with 2-mercaptoethanol and equal quantities of protein (500 ng/lane), separated under reducing conditions by SDS-PAGE and transferred onto nitrocellulose membranes using a transblot apparatus (Bio-Rad). After pre-incubation in blocking buffer (5% skim milk powder in PBS, 0.1% Tween 20) for 1 h, membranes were incubated with primary antibodies at 4 °C overnight, washed three times with blocking buffer, and then further incubated with alkaline phosphatase-conjugated secondary antibodies for 2 h at AT. After further washing in 0.1 M Tris, pH 9.5, containing 0.05 M MgCl_2_ and 0.1 M NaCl, specific antigen-antibody complexes were detected using nitro blue tetrazolium and 5-bromo-4-chloro-3-indoylphosphate (p-toluidine salt; Pierce). Specific β-actin antibody was used for the internal control to normalize the sample amounts.

### 2.10. Statistical Analysis

All experiments were performed three times as individual experiments with three replicates. For statistical analysis, a Wilcoxon–Mann–Whitney test was applied. Score values for image quality and presence of artifacts were compared for each sequence.

Data were shown as mean values ± SD or SEM, and were compared by one-way or two-way analysis of variance (ANOVA), if the normality test passed (Kolmogorov–Smirnov test). Otherwise, the Kruskal–Wallis ANOVA on ranks was used. A *p* value of < 0.05 was considered to establish statistically significant differences.

## 3. Results

The purpose of this study was to investigate the effect of resveratrol on regulating colorectal cancer cell (CRC) invasion and metastasis through regulation of focal adhesion molecules. We used two well-characterized colorectal cancer cell lines (HCT116 and SW480) in monolayer, as well as 3D alginate tumor microenvironment culture to address this question. The concentration of ethanol or DMSO applied in our study had no suppressive effect on CRC cell viability.

### 3.1. Resveratrol Alters Phenotype of CRC Cells Similar to FAK-Inhibitor or Cytochalasin D

**Figure 1:**
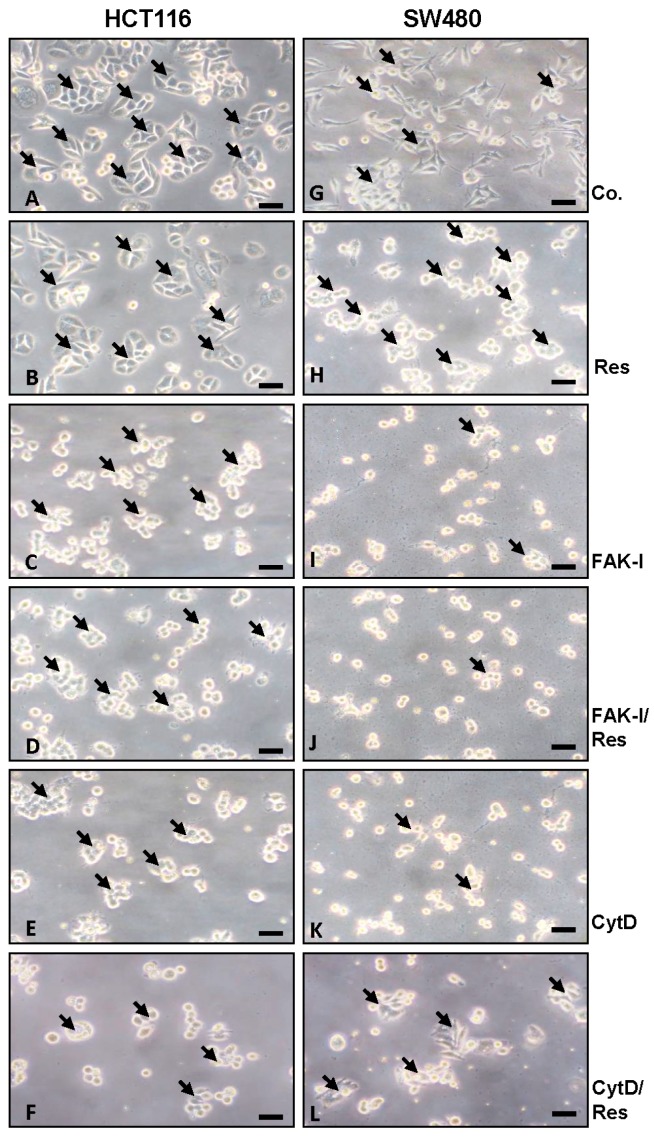
Effects of resveratrol and/or specific FAK-inhibitor and cytochalasin D on the phenotype of CRC cells. HCT116 and SW480 cells were cultured in monolayer and either left untreated alone (**A**,**G**) or were treated with 5 µM resveratrol alone (**B**,**H**), 10 µM FAK-I (PF-562271) (**C**,**I**), 0.1 µg/mL CytD (**E**,**K**) or a combination of 5 µM resveratrol and either 10 µM FAK-I (PF-562271) (**D**,**J**) or 0.1 µg/mL CytD (**F**,**L**) for 12 h and images were captured with a light microscope as described in materials and methods. Magnification 400×; bar = 30 nm.

Since cytoskeletal remodeling is critical for cancer cell invasion and migration [8], we first evaluated whether resveratrol alters colorectal cancer cell morphology cytoskeleton organization. HCT116 and SW480 cells in monolayer cultures were left untreated, treated with 5 µM resveratrol, with 10 µM FAK-I (PF-562271), with 0.1 µg/mL CytD alone, or a combination of 5 µM resveratrol and either 10 µM FAK-I or 0.1 µg/mL CytD for 12 h. As shown in Figure 1, in HCT116 untreated control, cultures evenly distributed cell colonies (arrows) with overall epithelial cell morphology and close cell-to-cell contacts were observed (Figure 1A). In contrast to HCT116, untreated SW480 cells showed a more mesenchymal morphology, less colony formation and fewer tight cell-to-cell contacts. The SW480 cells demonstrated a flattened shape and numerous microvilli-like cytoplasmic processes (Figure 1G). However, in both cell lines treatment with resveratrol significantly increased epithelial morphology, enhanced tight cell-to-cell contact and showed that most of the cells developed rounded morphology (Figure 1B,H). In contrast, in both cell lines treatment with FAK-I or CytD led to rounding of the cells and dissolution of the epithelial cell cluster with clear residues of adhesion molecules visible. Furthermore, the remnants of adhesive contacts and many detached cells were observed (Figure 1C,E,I,K). Combined treatment with resveratrol markedly increased the observed effects of FAK-I or CytD mono-treatment in HCT116 and SW480 (Figure 1D,F,J,L). Specially, it was observed that cells maintained attachment better than without resveratrol treatment and more cell clusters were formed.

### 3.2. Resveratrol Suppresses Cancer Cell Viability Similar to FAK-Inhibitor or Cytochalasin D

**Figure 2:**
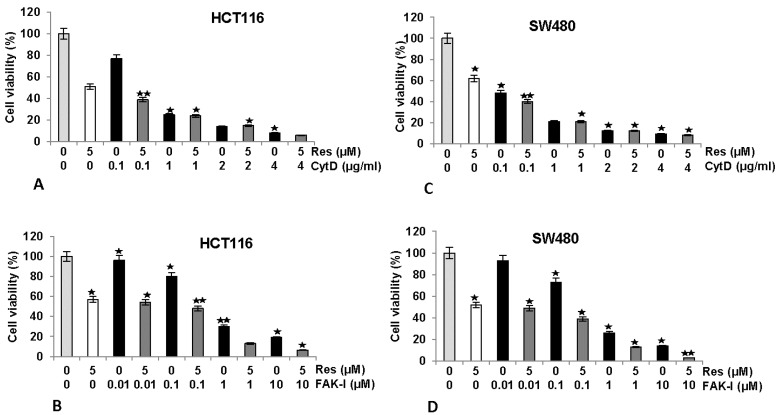
Effects of resveratrol and/or specific FAK-inhibitor and cytochalasin D on the proliferation of CRC cells. MTT assay showed inhibition of cell proliferation and viability in HCT116 (A,B) and SW480 (C,D) cell lines treated with resveratrol and/or different concentrations of CytD (0.1, 1, 2, and 4 µg/mL) or FAK-Inhibitor (PF-562271: 0.01, 0.1, 1, and 10 µM) cultured in monolayer for 12 h. Data shown are representative of three independent sets of experiments. Values were compared with the control and statistically significant values with *p* < 0.05 are designated by an asterisk (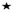) and *p* < 0.01 are designated by two asterisks (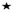

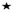
).

We next investigated the effects of FAK-I or CytD and/or resveratrol on cell viability by performing a MTT assay in HCT116 and SW480 cells (Figure 2). Serum-starved HCT116 and SW480 in monolayer culture were either left untreated, treated with 5 µM resveratrol or different concentrations of CytD (0.1, 1, 2, and 4 µg/mL), different concentrations of FAK-I (PF-562271: 0.01, 0.1, 1, and 10 µM) alone or co-treated with 5 µM resveratrol and the indicated concentrations of either CytD or FAK-I for 12 h. As shown for MTT results in Figure 2, cell viability in HCT116 was markedly reduced by 42–50% with resveratrol treatment compared to controls (Figure 2A,B). Furthermore, significant dose-dependent decreases of 23%, 77%, 91% and 95% in cell viability of HCT116 treated with 0.1, 1, 2 and 4 µg/mL CytD, were observed, respectively. Cell viability was further decreased by 62%, 78%, 90% and 97% in combination treatment of resveratrol and 0.1, 1, 2 and 4 µg/mL CytD, respectively (Figure 2A). Treatment with FAK-I induced significant dose-dependent decreases of 4%, 21%, 71% and 77% in cell viability of HCT116 treated with 0.01, 0.1, 1 and 10 µM FAK-I, respectively (Figure 2B). In SW480 cells, resveratrol treatment significantly reduced cell viability by 38–48% (Figure 2C,D). Significant dose-dependent decreases of 51%, 81%, 84% and 90% in cell viability of SW480 treated with 0.1, 1, 2 and 4 µg/mL CytD, respectively, were observed (Figure 2C). Cell viability in cells treated with a combination of resveratrol and 0.1, 1, 2 and 4 µg/mL CytD significantly decreased to 60%, 81%, 85% and 93%, correspondingly (Figure 2C). Treatment with 0.01, 0.1, 1 and 10 µM FAK-I markedly decreased cell viability of SW480 dose-dependent by 8%, 25%, 75% and 85%, respectively (Figure 2D). Interestingly, it was noted that co-treatment with resveratrol and 0.01, 0.1, 1 and 10 µM FAK-I markedly suppressed decrease of 51%, 61%, 86% and 96%, respectively, in cell viability of SW480 compared to mono-FAK-I treatment (Figure 2D).

### 3.3. Resveratrol-Induced Sirt1 Expression Is Mediated by Focal Adhesion Kinase and Cytoskeletal Proteins in CRC Cells Monolayer Culture as Revealed by Immunofluorescence Microscopy

**Figure 3:**
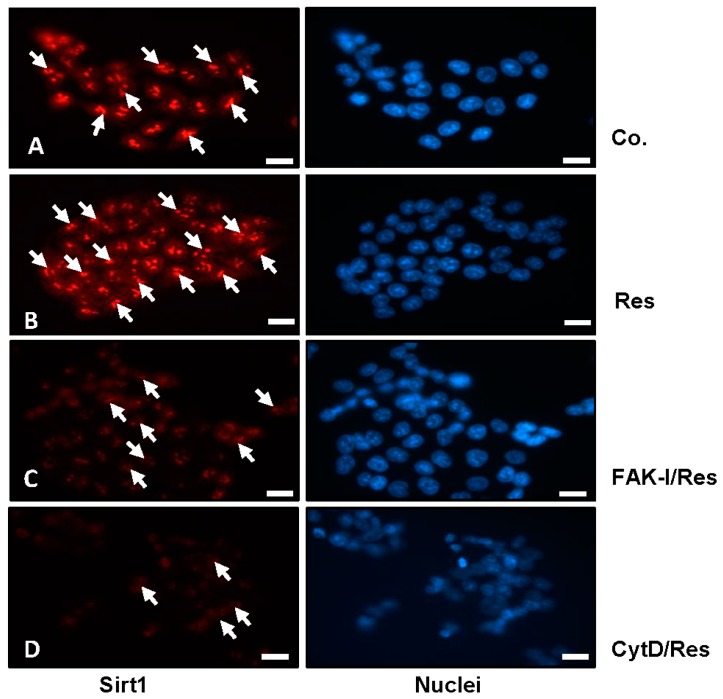
Effects of resveratrol and/or specific FAK-inhibitor and cytochalasin D on the Sirt1 expression of CRC cells. Serum-starved HCT116 cells were cultured on glass plates in monolayer and either left untreated alone (**A**), or were treated with 5 µM resveratrol alone (**B**), or a combination of 5 µM resveratrol and either 10 µM FAK-I (PF-562271) (**C**) or 0.1 µg/mL CytD (**D**) for 12 h and subjected to immunofluorescent labeling with anti-Sirt1 and nuclear counterstaining with DAPI. Magnification 400×; bar = 30 nm

The histone deacetylase Sirt1 is a major subcellular target of resveratrol [39,40] and is a major target for resveratrol-mediated suppression of colorectal cancer cell growth and metastasis [30,41]. Therefore, we investigated the role of FAK or cytoskeletal organization on the resveratrol-induced up-regulation of Sirt1 expression (arrows) and of the possible associated mechanism in colorectal cancer cells. Serum-starved HCT116 and SW480 cultures were left untreated, treated with 5 µM resveratrol or treated with resveratrol (5 µM), and co-treated with 10 µM FAK-I (PF-562271) or with 0.1 µg/mL CytD for 12 h and subjected to immunofluorescence labeling with primary antibodies for Sirt1 and rhodamine-coupled secondary antibodies. Counterstaining was performed with DAPI to visualize the cell nuclei. As shown in Figure 3B, resveratrol alone significantly upregulated the expression of Sirt1 protein and nuclear localization compared to the controls (Figure 3A). However, the resveratrol-induced expression of Sirt1 protein in the nuclei of HCT116 was significantly decreased by incubation with FAK-I and CytD (Figure 3C,D), indicating that FAK and cytoskeletal signaling proteins, at least in part, are some of the main target proteins of resveratrol during the resveratrol-induced anti-tumor effect in CRC cells.

### 3.4. Resveratrol-Induced Formation of Focal Adhesions in CRC Cells Are Inhibited by FAK-Inhibitor or Cytochalasin D

**Figure 4:**
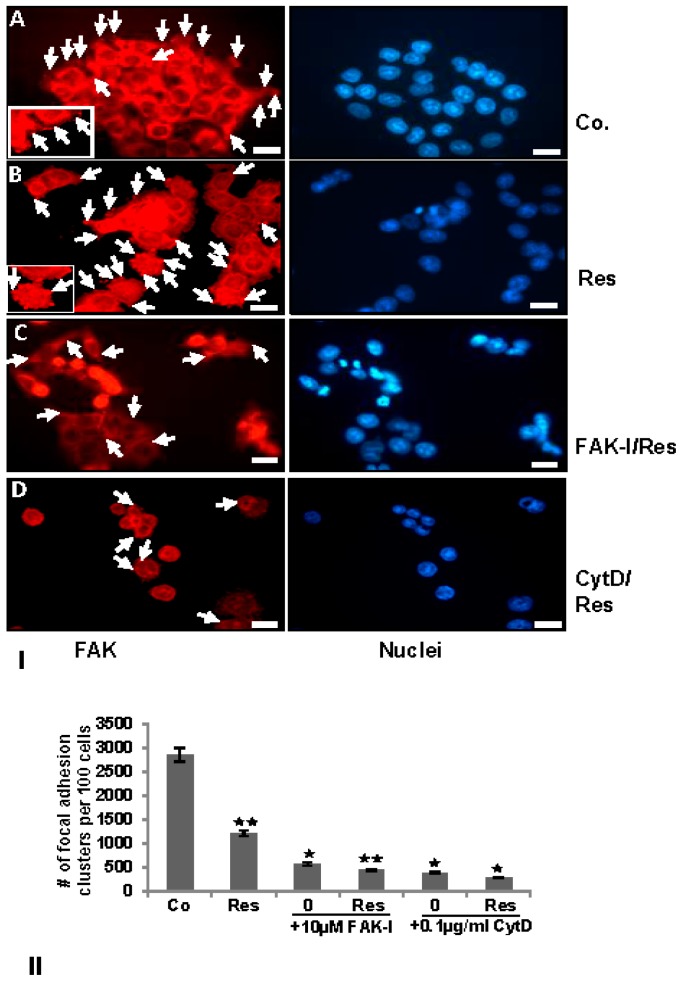
Effects of resveratrol and/or specific FAK-inhibitor and cytochalasin D on the focal adhesion clusters of CRC cells. (I) Serum-starved HCT116 were either left untreated (**IA**,**II**), were treated with 5 µM resveratrol alone (**IB**,**II**), 10 µM FAK-I (PF-562271) (**II**), 0.1 µg/mL CytD (**II**) or a combination of 5 µM resveratrol and either 10 µM FAK-I (PF-562271) (**IC**,**II**) or 0.1 µg/mL CytD (**ID**,**II**) for 12 h. Immunofluorescent labeling with antibodies against FAK and counterstaining with DAPI to visualize cell nuclei was performed on HCT116 cells in monolayer. (II) To quantify the amount of focal adhesion, 800–1000 cells from 10 microscopic fields were counted. The examination was performed in triplicate, and the results are provided as the mean values with S.D. from three independent experiments. Values were compared with the control and statistically significant values with *p* < 0.05 are designated by an asterisk (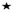) and *p* < 0.01 are designated by two asterisks (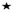

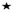
). Magnification 400×; bar = 30 nm.

FAK has been shown to be involved in cancer cell survival and invasion [18] and on the other hand resveratrol has been shown to suppress cancer cell migration/invasion [26] and inhibition of phosphorylation of FAK in pancreatic cancer [33,34,35]. To examine the effects of resveratrol on the FAK expression in colorectal cancer cells, serum-starved HCT116 cultures were treated as described above and subjected to immunolabeling with anti-FAK. Strong staining for FAK was observed in untreated controls in HCT116 (Figure 4). In control cell cultures, a distribution of FAK positive labeled focal adhesion points (arrows) was observed on the cytoplasmic membranes (Figure 4I(A), arrows and inset). Contrary to this, resveratrol reduced FAK labeling to a basal level, but interestingly, induced formation of focal adhesion clusters, which were strongly positively stained for FAK (Figure 4I(B), arrows and inset). The combination treatment with resveratrol and with FAK-I or CytD markedly reduced FAK staining in both HCT116 (Figure 4I(C)). Quantification of focal adhesion points confirmed that resveratrol, FAK-I, CytD or combination treatment markedly decreased the number of focal adhesion clusters compared to untreated control cultures in HCT116 (Figure 4II).

### 3.5. Specific Inhibitors against FAK Lead to Increased Apoptosis in CRC Cells

**Figure 5:**
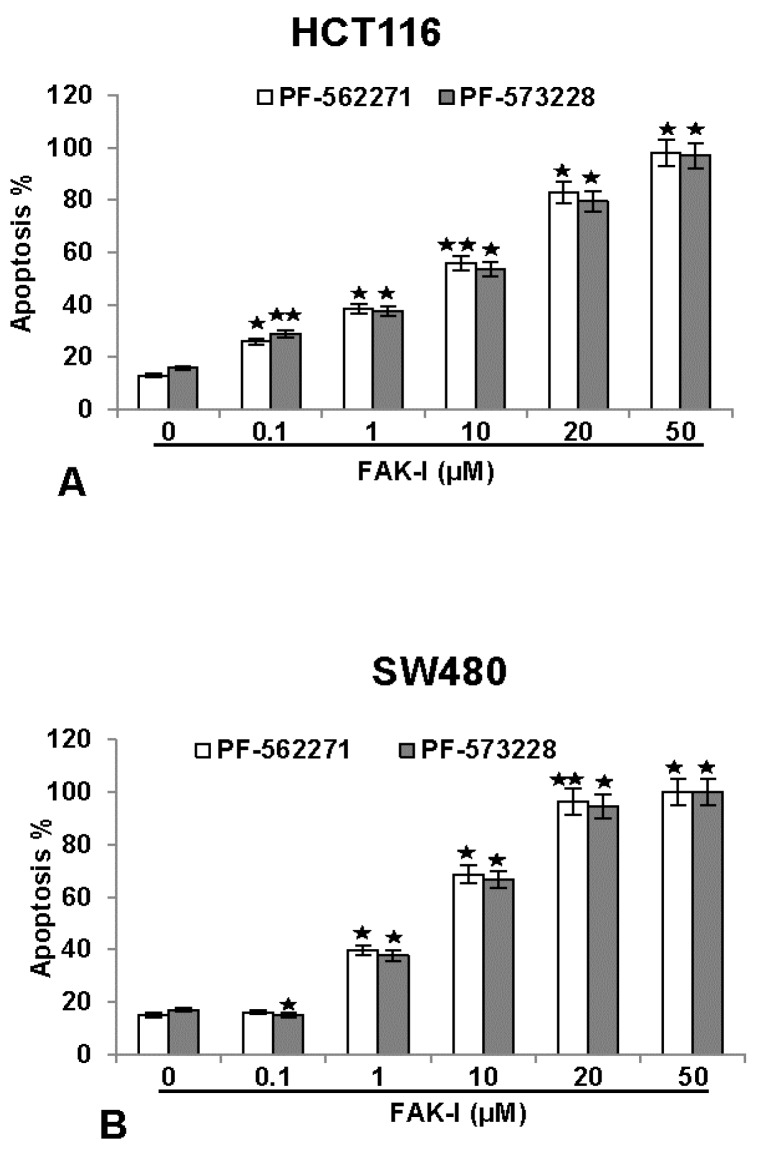
Effects of specific FAK-inhibitors (PF-562271 and PF-573228) on the apoptosis of CRC cells. Serum-starved HCT116 (**A**) and SW480 (**B**) were treated with different concentrations of FAK-inhibitors (PF-562271, PF-573228: 0, 0.1, 1, 10, 20 and 50 µM) for 12 h. Monolayer cultures were fixed with methanol and nuclear staining assay (DAPI) was performed to reveal nuclear apoptotic changes. Quantification of apoptotic cells was performed by scoring 800–1000 cells from 10 different microscopic fields. One of three independent experiments is shown. Values were compared to the control and statistically-significant values with *p* < 0.05 are designated by an asterisk (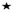); *p* < 0.01 by two asterisks (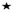

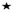
).

To investigate the effects of two different FAK inhibitors (PF-562271 and PF-573228) on the growth rate inhibition and the levels of apoptosis on colorectal cancer cells, HCT116 and SW480 were treated with different concentrations of FAK-I (0.1, 1, 10, 20 and 50 µM) for 12 h and a nuclear staining assay with DAPI (4, 6-Diamidino-2-phenylindole, Sigma) was performed. This fluorescence-based staining method reveals apoptotic bodies containing nuclear fragmentation and chromatin condensation in apoptotic cells. The number of apoptotic nuclei was quantified by scoring 800–1000 cells from 10 different microscopic fields. As shown in Figure 5, the number of apoptotic nuclei in control cultures of both HCT116 and SW480 ranged from 8–15%. The number of apoptotic nuclei markedly increased in both cell lines by FAK inhibitor treatment. A significant dose-dependent effect was observed in HCT116 treated with 0.1, 1, 10, 20 and 50 µM PF-562271 with an apoptotic increase of 23%, 39%, 46%, 82% and 98%, as well as with 0.1, 1, 10, 20 and 50 µM PF-573228 with an apoptotic increase of 30%, 38%, 41%, 79% and 96%, respectively (Figure 5A). In SW480 cells, a low concentration of 0.1 µM PF-562271 or PF-573228 resulted in 15% and 13% apoptotic cells, respectively, similar to untreated controls. Contrary to this, a significant dose-dependent increase in apoptosis was observed in SW480 cells treated with 1, 10, 20 and 50 µM PF-562271 with an apoptotic increase of 40%, 69%, 97% and 100%, as well as with 1, 10, 20 and 50 µM PF-573228 with an apoptotic increase of 38%, 65%, 95% and 100%, respectively (Figure 5B). These results demonstrate that FAK-I shows similar levels of apoptotic induction in both cell lines.

### 3.6. Resveratrol Potentiates FAK-Inhibitor- and Cytochalasin D-Induced Activation of Caspase-3 in CRC Cells Monolayer Culture as Revealed by Immunofluorescence Microscopy

**Figure 6:**
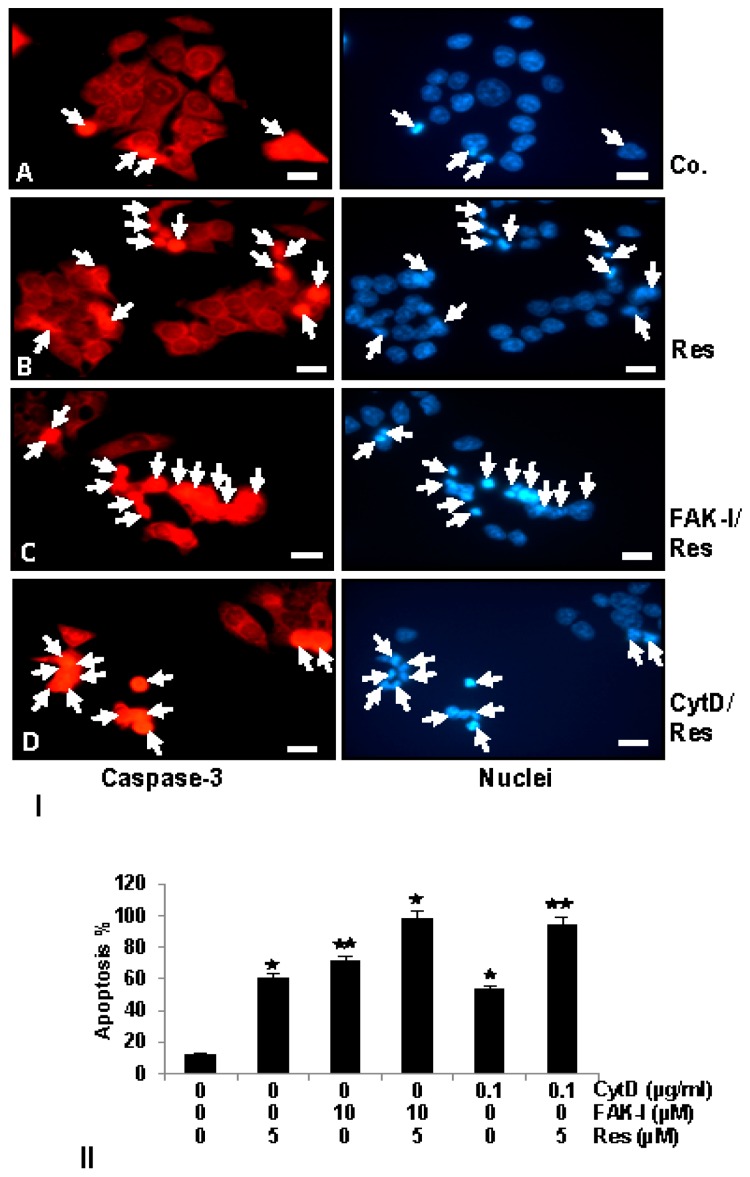
Effects of resveratrol and/or specific FAK-inhibition and cytochalasin D on the apoptosis of CRC cells. (**I**) Serum-starved HCT116 cell lines in monolayer culture were either left untreated (**IA**,**II**), or were treated with 5 µM resveratrol alone (**IB**,**II**), 10 µM FAK-I (PF-562271) (**II**), 0.1 µg/mL CytD (II) or a combination of 5 µM resveratrol and either 10 µM FAK-I (PF-562271) (**IC**,**II**) or 0.1 µg/mL CytD (**ID**,**II**) for 12 h. Immunofluorescent labeling with antibodies against caspase-3 followed by incubation with rhodamine-coupled secondary antibodies and counterstaining with DAPI to visualize cell nuclei. Images shown are representative of three different experiments; (**II**) Quantification of apoptotic cells was performed by scoring 800–1000 cells from 10 different microscopic fields. One of three independent experiments is shown. Values were compared to the control and statistically-significant values with *p* < 0.05 are designated by an asterisk (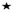); *p* < 0.01 by two asterisks (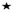

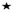
). Magnification 400×; bar = 30 nm.

To examine the mechanisms by which resveratrol and/or FAK-I or CytD inhibit cell viability and induce apoptosis of CRC cells, we analyzed and compared their effect on the rate of the activation levels of caspase-3, which induces the apoptotic pathway in cells [42,43]. Therefore, the amount of apoptosis induced by resveratrol, FAK-I, CytD and their combined treatment in HCT116 (Figure 6I) was confirmed by investigating the activation of caspase-3 with immunofluorescent labeling and induction of morphological nuclear changes with DAPI nuclear staining (arrows) (Figure 6II). In untreated control cultures of HCT116 (Figure 6I(A)) only small amounts of caspase-3 positive stained cells (arrows) could be detected. In contrast to this, treatment with resveratrol, markedly induced caspase-3 expression in colorectal cancer cells (Figure 6I(B)). Interestingly, a significant increase of caspase-3 was observed in the combined treatment of resveratrol with either FAK-I or CytD (Figure 6I(C,D)).

Apoptosis induction by caspase-3 activation was confirmed by examining morphological nuclear changes indicative of apoptosis with DAPI nuclear staining (Figure 6II). The number of apoptotic nuclei was quantified by scoring 800–1000 cells from 10 different microscopic fields. Nuclear staining revealed that untreated control HCT116 (Figure 6II) cell cultures displayed an overall normal nuclear size with around 12% of apoptotic nuclei in the cell culture. In contrast, in cells treated with resveratrol, a significant increase of 59% apoptotic cells in HCT116 with nuclear morphological changes such as pyknosis and chromatin condensation was observed. Significant dose-dependent increases in apoptosis was observed in HCT116 treated with 10 µM FAK-I (PF-562271), with an apoptotic rate of 70%, and with 0.1 µg/mL CytD, with an apoptotic rate of 56% (Figure 6II). Furthermore, a significant increase in fragmented nuclei and apoptotic features was observed by combined treatment of resveratrol and either 10 µM FAK-I (51%) or 0.1 µg/mL CytD (94%) compared to the treatment of only resveratrol, FAK-I or CytD.

### 3.7. Resveratrol Potentiates FAK-Inhibitor- and Cytochalasin D-Inhibited Invasion of CRC Cells in 3D Alginate Tumor Microenvironment Culture

**Figure 7:**
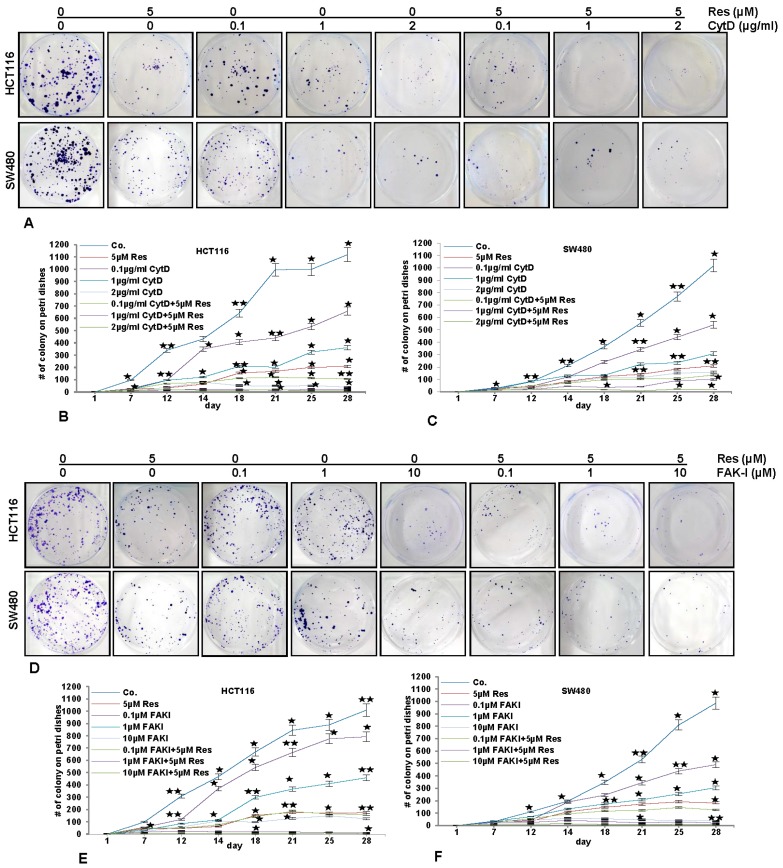
Effects of resveratrol and/or specific FAK-inhibition and cytochalasin D on the migration of CRC cells in alginate culture. (**A**–**C**) Serum-starved HCT116 and SW480 cell lines in alginate beads (1 × 10^6^) were either left untreated or treated with 5 µM resveratrol alone, different concentrations of CytD (0.1, 1, and 2 µg/mL) or co-treated with 5 µM resveratrol and the indicated concentrations of CytD and emigrated spheroids evaluated by toluidine blue staining after 28 days; (**D**–**F**) Serum-starved HCT116 and SW480 cell lines in alginate beads (1 × 10^6^) were either left untreated or treated with 5 µM resveratrol alone, different concentrations of FAK-I (PF-562271: 0.01, 0.1, 1, 10 µM) or co-treated with 5 µM resveratrol and the indicated concentrations of FAK-I and emigrated spheroids evaluated by toluidine blue staining after 28 days. The values given are the means ± standard errors of the mean of three replicates. One of three independent experiments is shown. Values were compared with the control and statistically significant values with *p* < 0.05 are designated by an asterisk (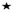); *p* < 0.01 by two asterisks (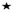

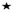
).

To investigate the effect of resveratrol, FAK-I and CytD on cell motility and invasion capacity through the 3D tumor microenvironment, HCT116 and SW480 cells were cultured in an alginate-based tumor microenvironment, treated as described in detail in material and methods and the capacity of migration and invasion was determined through the evaluation of the colony formation with toluidine blue staining after 10 days (Figure 7).

As shown in Figure 7, treatment of the CRC cells (HCT116 and SW480) with resveratrol (5 µM) alone significantly blocked (*p* < 0.05) the migration rate of HCT116 (Figure 7A,B-D,E) and SW480 (Figure 7A,C-D,F) cells through the alginate-based matrix after an incubation time of 28 days by 82% and 83%, respectively, compared to untreated cells. Treatment of CRC cells with CytD at 0.1, 1, and 2 µg/mL inhibited the migration of HCT116 through the alginate matrix by 42%, 68.2% and 95.5%, respectively (Figure 7A,B), and of SW480 (Figure 7A,C) by 51%, 73.6% and 86.4%, respectively. Additionally, the combined treatment of resveratrol (5 µM) and CytD (0.1, 1, and 2 µg/mL) was much more effective in comparison to the individual compound, enhancing invasion-inhibition ability of HCT116 to 91%, 98.2% and 99.1%, respectively (Figure 7A,B), and of SW480 to 89%, 91% and 97.3%, respectively (Figure 7A,C).

The treatment of CRC cells with FAK-I (PF-562271) (0.1, 1, 10 µM) inhibited migration of HCT116 through the alginate matrix by 22%, 58% and 88%, respectively (Figure 7D,E) and of SW480 by 49.5%, 72% and 96%, respectively (Figure 7D,F). Additionally, the combined treatment of resveratrol and FAK-I (0.1, 1, and 10 µM) was more effective than in individual treatment, as the invasion-inhibition ability was enhanced in HCT116 to 85%, 99.6% and 99.8%, respectively (Figure 7D,E), and in SW480 to 88.5%, 98% and 99.5%, respectively (Figure 7D,F).

### 3.8. Resveratrol-Mediated Anti-Tumorigenic Effects Are Potentiated by Inhibition of FAK and Cytoskeletal Proteins

**Figure 8:**
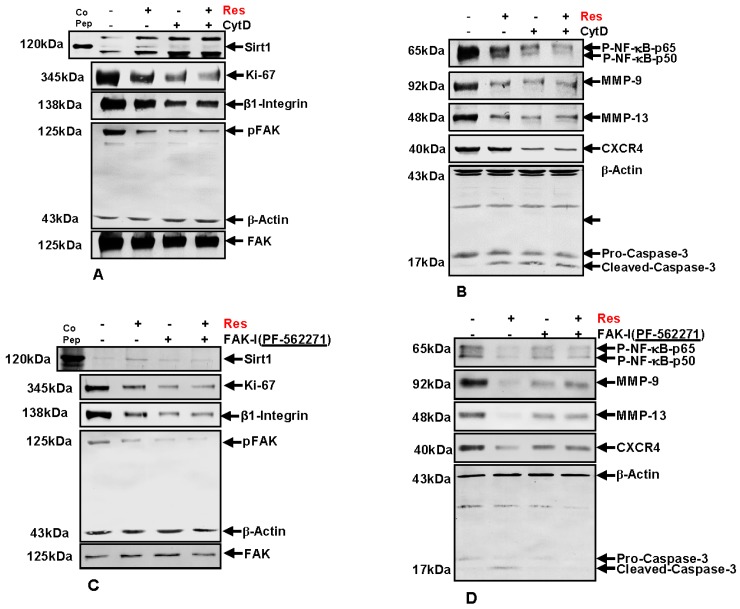
Effects of resveratrol and/or specific FAK-inhibitor and cytochalasin D on Sirt1; adhesion, proliferation, invasion, and metastatic gene products; and NF-κB signaling pathway in CRC cells. Serum-starved HCT116 cell line in alginate beads (1 × 10^6^) were either left untreated or treated with: 5 µM resveratrol alone or 0.1 µg/mL CytD (**A**,**B**); 10 µM FAK-I (PF-562271) (**C**,**D**); or co-treated with 5 µM resveratrol and either the indicated concentration of: CytD (**A**,**B**); or FAK-I (**C**,**D**) for 10 days. Whole cell lysates were fractionated and analyzed by Western blotting using antibodies against: Sirt1, Ki-67, β1-integrin, FAK, and p-FAK (**A**,**C**); and p-NF-κB-65/50, MMP-9, MMP-13, CXCR4, and caspase-3 (**B**,**D**). Western blots shown are representative of three independent experiments. The housekeeping protein β-actin served as a positive loading control in all experiments.

Next, to investigate the underlying mechanism of FAK signaling and cytoskeletal organization on the resveratrol-induced anti-carcinogenic effects in colorectal cancer cells, immunoblotting was performed. HCT116 (Figure 8) cells were cultured in tumor microenvironment alginate beads as described in materials and methods and either left untreated, or were treated with 5 µM resveratrol alone, 0.1 µg/mL CytD, 10 µM FAK-I (PF-562271) or co-treated with 5 µM resveratrol and either the indicated concentration of CytD or FAK-I for 10 days.

### 3.9. Resveratrol-Mediated Sirt1 Up-Regulation Is Suppressed by FAK Inhibitor or by Cytochalasin D

First, we investigated whether resveratrol-induced up-regulation of Sirt1 expression and the associated anti-proliferative effects in CRC cells are dependent on FAK and/or cytoskeletal signaling proteins. Total protein lysates were subjected to immunolabeling with Sirt1 and Ki67 antibodies (Figure 8A,C). As demonstrated by Western blot analysis, treatment with resveratrol up-regulated the expression of Sirt1 protein and significantly suppressed Ki67 compared to untreated cultures.

Destabilization of actin cytoskeleton by CytD or by inhibition of FAK with FAK inhibitor (PF-562271) markedly suppressed resveratrol-induced Sirt1 up-regulation in HCT116. Further, marked Ki67 suppression was observed in CytD, FAK-I or the combinational treatment (Figure 8A,C). These results highlight that, at least in part, FAK has a functional role in resveratrol-Sirt1-mediated anti-tumorigenic signaling in CRC cells.

### 3.10. Resveratrol-Induced Suppression of β1-Integrin and FAK Is Potentiated by FAK Inhibition and Cytochalasin D

It has been shown that FAK participates in integrin mediated signaling functions, thereby enabling cell adhesion signals which stimulate reorganization of the cytoskeleton [11,12], and resveratrol has been shown to decrease the levels of β1-integrins and inhibit phosphorylation of FAK in several cancer cell lines [32,33,34,35]. Therefore next, we investigated the effect of resveratrol on Integrin and FAK activation in CRC cells. HCT116 cells were treated as described above and Western blotting performed with Integrin, FAK and p-FAK antibodies (Figure 8A,C). As demonstrated in Figure 8, resveratrol treatment markedly suppressed Integrin expression and activation of FAK in CRC cells compared to untreated controls (Figure 8A,C). Further, treatment with CytD alone or in combination with resveratrol had a suppressive effect on Integrin expression and activation of FAK. Interestingly, marked down-regulation of Integrin expression and FAK phosphorylation was observed in individual treatment with CytD or FAK-I or in co-treatment with resveratrol, but had no such effect on basal, inactivated FAK levels. Taken together, these results support a functional role for Integrin-FAK-mediated signaling and cytoskeletal signaling proteins as target proteins, at least in part of resveratrol mediated anti-tumorigenic effects in CRC cells.

### 3.11. Resveratrol-Potentiates FAK-Inhibitor- and Cytochalasin D-Induced Suppression of NF-κB Activation in CRC Cells

It has been previously shown that resveratrol has a functional role in suppression of the NF-κB signaling pathway in CRC cells [26]. To elucidate whether suppression of NF-κB signaling by resveratrol is modulated by FAK activity or cytoskeletal signaling proteins, HCT116 and SW480 cells were treated as described above and immunoblotting was performed with anti-NF-κB-p50 and anti-NF-κB-p65 (Figure 8B,D). Immunoblotting analysis demonstrated that resveratrol treatment significantly suppressed activation of NF-κB compared to control cultures (Figure 8B,D). Further, phosphorylation of NF-κB subunits (p50/p65) was markedly suppressed by CytD or FAK inhibition in HCT116 (Figure 8B,D). Interestingly, a synergistic effect on suppression of NF-κB activation was observed in the combination treatment with either CytD or FAK inhibitor. Overall, these results indicate that resveratrol-induced suppression of NF-κB activation is at least in part dependent on FAK and cytoskeletal signaling proteins.

### 3.12. Resveratrol-Potentiates FAK-Inhibitor- and Cytochalasin D- Induced Suppression of NF-κB-Dependent Gene End Products Involved in Invasion, Metastasis and Apoptosis

To further characterize the role of FAK and cytoskeletal signaling proteins on resveratrol-mediated suppression of malignancy and metastatic ability in HCT116 cells, protein expression of tumor metastasis associated promoting factors influencing tumor cell invasion, metastasis and apoptosis were investigated. CRC cells were treated as described above and Western blotting was performed for anti-MMP-9, -MMP-13, -CXCR4 and -cleaved-caspase-3 (Figure 8B,D). Compared to untreated control cultures resveratrol significantly suppressed MMP-9, MMP-13 and CXCR4 and markedly up-regulated activated Caspase-3 in CRC cells (not shown). Interestingly, these effects were significantly stronger in resveratrol mono-treatment compared to CytD, FAK-I or the combinational treatment. These results highlight that resveratrol-induced suppression of malignancy, metastatic ability and induction of apoptosis in CRC cells is, at least in part, mediated by FAK and cytoskeleton signaling proteins.

## 4. Discussion

Our data reveal that resveratrol indeed suppresses CRC proliferation and invasion by up-regulation of Sirt1 activity, inhibition of NF-κB-mediated inflammatory pathway and suppression of focal adhesion kinase (FAK) activity, resulting in a loss of focal adhesion molecules, a planar surface of the CRC cells and an increase in apoptosis.

Despite major advances in oncology, colorectal cancer (CRC) remains one of the major causes of cancer-related morbidity and mortality worldwide [1]. Although treatment options range from radiotherapy, surgery and chemotherapy to combination treatment, chemotherapy still offers the best chance to prevent invasion and metastasis. However, most chemotherapies fail to effectively combat metastatic cancer and have high prevalence of recurrence, chemoresistance and are toxic and expensive.

Therefore, new pharmacologically effective and safe therapeutic approaches that may prevent the initial invasion of cancer cells from primary tumors and thus prevent metastasis are urgently sought. Natural compounds such as resveratrol have gained much attention for preventive and therapeutic strategies in cancer treatment including colorectal cancer [44,45]. Resveratrol (3,5,4′-trihydroxy-trans-stilbene), found in more than 70 plant species, has shown to exhibit both chemopreventive and chemotherapeutic potential [20,21,22]. We have shown in this study that resveratrol indeed altered the cell phenotype of two CRC cell lines HCT116 and SW480, by inducing cell cluster formation, encouraging cell attachment and reducing cell viability. Indeed, in a study from our own laboratory we could previously show that resveratrol affects multiple cell adhesion molecules, desmosomes, tight, gap junctions and signaling molecules involved in cell proliferation, invasion and metastasis [26].

Further, we found that treatment of the CRC cell lines with an inhibitor to FAK or with CytD markedly promoted cellular attachment and rather more cell clusters were observed. Indeed, it has been previously shown that cancer cell invasion (migration) is an essential initial step of spontaneous carcinogenesis and metastasis and to understand more detailed this important mechanism may be useful for the treatment/prevention of CRC. Focal adhesion kinase (FAK) is an intracellular tyrosine kinase that regulates cellular adhesion, control of cell-extracellular interactions such as cell spreading, migration, metastasis, motility, cancer stem cell self-renewal and survival in many types of cancers and FAK is overexpressed in a number of tumors including CRC [18]. As FAK is a non-receptor tyrosine kinase that is activated in response to cell adhesion and transduces extracellular signals through tyrosine phosphorylation onto various intracellular molecules in both growth factor dependent and adhesion-dependent manner, it is fundamentally involved in modulating cancer progression and metastasis [8]. Interestingly, in several cancers, including breast and ovarian carcinomas, genetic tumor profiling has revealed both FAK DNA amplification (*PTK2* gene at 8q24.3) and elevated FAK mRNA levels [19]. Indeed, activation of FAK has been shown to be high in metastatic aggressive tumors and is correlated with poor clinical outcome [8].

Cytochalasins are a class of fungal metabolites affecting a wide variety of motile functions of eukaryotic cells [46]. CytD has been described to de-stabilize, disorganize and alter the actin cytoskeleton [47,48]. Here we could demonstrate that FAK inhibition and CytD additionally dose-dependently reduced CRC HCT116 and SW480 cell viability, induced cell cluster formation and encouraged cell attachment similar to resveratrol treatment. Indeed, it has been previously shown that resveratrol or FAK-inhibition suppresses cancer cell viability and survival [8,26,30,41]. These observed effects of resveratrol, FAK- inhibition and CytD in HCT116 and SW480 CRC cells highlight the involvement of cytoskeletal signaling molecules in resveratrol-mediated anti-tumorigenic effects.

We have further shown that resveratrol up-regulates Sirt1 expression and suppresses FAK expression in HCT116 and SW480 CRC cells. It has been previously suggested that resveratrol acts as a multi-targeted agent in cancer as it modulates several cellular signaling pathways involved in cancer cell survival, proliferation and metastasis [23,24,25,26]. Indeed, it has been previously shown that the nucleus related NAD^+^ histone deacetylase class III sirtuin 1 (Sirt1), is a major target for resveratrol [40]. Resveratrol provokes a structural conformational change in Sirt1, consequently resulting in an increased enzymatic activity [40]. Moreover, we could demonstrate previously that resveratrol-induced Sirt1 up-regulation is required for the resveratrol-mediated chemopreventive effects in colorectal cancer cells [30]. Further, combinational treatment of resveratrol with FAK-I or CytD suppressed resveratrol-induced Sirt1 activation and FAK. Interestingly, it has been shown that deletion of Sirt1 in bone marrow macrophages (BMMs) increased expression and acetylation of FAK, as well as NF-κB [49]. This highlights a potential role for FAK-Sirt1 signaling pathway in resveratrol-mediated anti-tumorigenic effects of resveratrol in CRC cells.

It has been previously described that inhibition of FAK impairs cancer development in preclinical and clinical trials [8]. Indeed, in our study, we could demonstrate a marked dose-dependent effect of specific FAK inhibitors (PF-562271 or PF-573228) on growth inhibition and induction of apoptosis in HCT116 and SW480 CRC cells. Both specific FAK inhibitors are ATP-competitive, reversible inhibitors of FAK. Indeed, it has been recently shown that inhibition of FAK, with specific FAK inhibitors, enhances chemosensitivity and suppresses invasion of cancer cells such as lung and breast carcinoma [50,51]. This underlines the important aspect of active FAK-signaling for the interaction between the CRC cells and their surrounding tumor microenvironment to control and regulate cancer progression and metastasis. It is well known that activation of caspase-3 induces the apoptotic pathway in cells [42,43]. We further demonstrated that indeed resveratrol alone activated caspase-3 and potentiated nuclear signs of apoptosis in CRC cells and this was markedly enhanced by combinational treatment of resveratrol with FAK-I or CytD. Furthermore, here we assessed a potential relationship between resveratrol and FAK-signaling pathway and investigated the combined potential to enhance and stimulate apoptosis in CRC cells. Interestingly, it has been recently shown that inhibition of FAK and CXCR4 and simultaneous treatment of lung carcinoma cells with doxorubicin potentiated their anti-tumorigenic effects [51]. These results highlight that FAK and cytoskeletal signaling molecules are, at least in part, one of the main target proteins for resveratrol-mediated anti-tumorigenic effects in CRC cells.

Several reports from our and other laboratories have reported that three dimensional culture models provide excellent in vitro culture conditions compared to two dimensional models, as they more adequately represent physiological conditions that exist in vivo [37,52,53] Indeed, recent reports from our laboratory have shown that alginate bead culture provides an excellent tumor microenvironment were the CRC cells can proliferate, form spheroids, migrate through the alginate matrix and finally form metastases [30,37]. Our data in this study indicate that resveratrol suppressed invasion (migration) and colony forming capacity of HCT116 and SW480 CRC cells in alginate bead tumor microenvironment culture, similar to FAK inhibitor or CytD. This is consistent with previous reports, which showed that resveratrol inhibits angiogenesis, proliferation and metastasis in different kinds of tumors [54,55,56]. Additionally, the anti-invasive and anti-metastases effect of resveratrol was potentiated in the combinational treatment of FAK-I and CytD. Cytoskeleton reorganization and remodeling is a critical step for cancer cell invasion and metastasis. In line with our results, FAK inhibition in a murine model with PF-562271 resulted in decreased tumor cell proliferation, migration and tumor-associated macrophages and fibroblasts [57]. The data presented here now underline the importance of participation of FAK and cytoskeletal signaling proteins in resveratrol mediated anti-invasive and anti-metastasis properties in CRC cells.

Further, we also found in alginate tumor microenvironment cultures of CRC cells that resveratrol-induced up-regulation of Sirt1, blocked CRC cell proliferation and concomitantly suppressed Integrin and activation of FAK. In contrast to this, FAK inhibitor or CytD alone did not up-regulate Sirt1 expression, but merely suppressed Integrin expression and activation of FAK. Resveratrol has been previously shown to suppress migration of cancer cells by modulation of integrin activity [31] and to decrease the levels of cell adhesion proteins and suppress expression of epithelial-to-mesenchymal-transition associated mediators α5β1 integrins and hyaluronic acid in ovarian cancer cell lines [32]. Interestingly, resveratrol has been further shown to decrease the levels of β1-integrins and at the same time inhibit phosphorylation of FAK in several cancer cell lines [32,33,34,35]. Further, it has been shown that FAK participates in integrin-mediated signaling functions, thereby enabling cell adhesion signals that stimulate reorganization of the cytoskeleton [11,12]. Indeed, it has been suggested that overexpression of β4-Integrins and FAK in colorectal cancer lead to subsequent FAK activation, essential for early steps of tumorigenesis [58].

Furthermore, we also found that resveratrol, FAK-I or CytD inhibited activation of NF-κB, suppressing NF-κB–dependent gene end-products involved in invasion (MMPs), metastasis (CXCR4) and activating those involved in apoptosis (cleavage of caspase-3). Interestingly, these observed effects of resveratrol were significantly potentiated by combinational treatment with either FAK-I or CytD. It has been previously reported that resveratrol inhibits IκB-kinase-β-mediated NF-κB activation and it is a potent natural activator of Sirt1 [27,28,29]. Indeed, in CRC cells, resveratrol-induced Sirt1 has a functional role in inhibiting activation of the NF-κB signaling pathway [26,30], wereby Sirt1 deacetylates the NF-κB-p65 subunit and thus attenuates NF-κB-mediated gene transcription and inflammation pathways [59]. Further, it has been shown that deletion of Sirt1 increased expression and acetylation of FAK, as well as NF-κB [49]. Moreover, it has been observed that FAK, besides its canonical roles as a cytoplasmic kinase downstream of integrin and growth factor receptor signaling, can also serve as a co-transcriptional regulator that alters a gene transcriptional activity by shuttling from focal adhesions to the nucleus to directly convey extracellular signals [60]. Further, FAK contains NF-κB and p53 binding sites, which regulate induction and inhibition of FAK transcription [61]. Transcription of FAK is regulated by binding of NF-κB to the *Ptk2* gene promoter and activation of NF-κB by TNF-α increases FAK transcriptional activation [61]. These results highlight the potential pharmacological use of resveratrol-induced Sirt1 pathway in targeting FAK and NF-κB in chronic inflammatory diseases including cancer.

## 5. Conclusions

**Figure 9:**
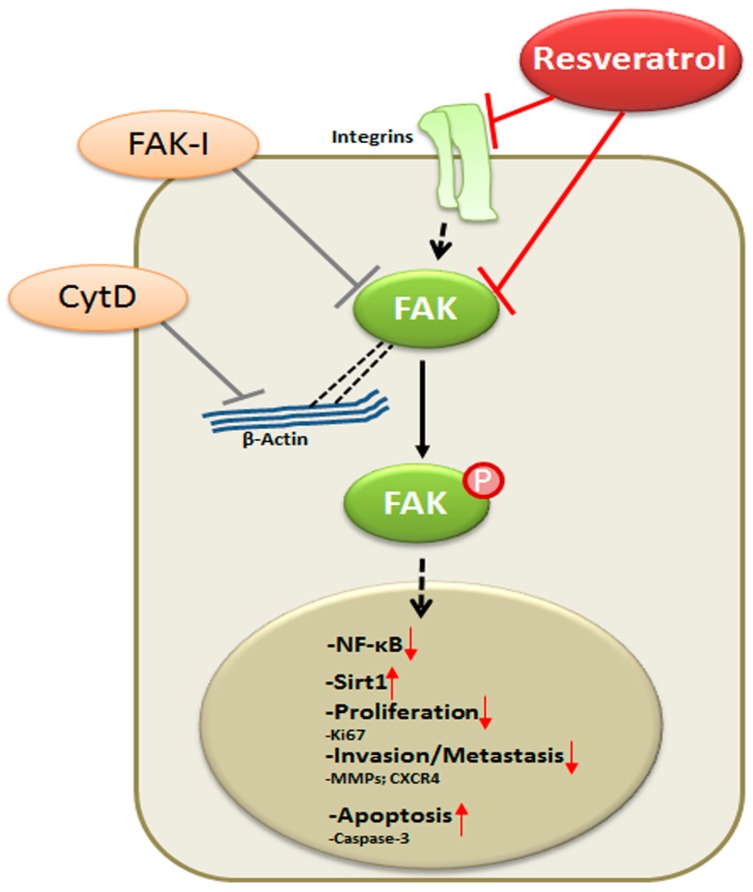
Schematic diagram shows resveratrol-mediated antitumor activity by modulation of focal adhesion kinase in colorectal cancer cells.

In conclusion, for the first time, we describe herein that the anti-cancerogenic mechanism of resveratrol is mediated, at least partially, by the inhibition of FAK in CRC cells. This results in suppression of early steps of cancerogenesis, with increased apoptosis and suppression of migration and metastasis in cancer cells (Figure 9). In addition, we could further demonstrate that FAK-inhibition suppresses resveratrol-induced Sirt1 activation. As Sirt1 is an important molecular target of resveratrol, it seems that the anti-cancerogenic effect induced by resveratrol-Sirt1-activation in CRC cells is mediated at least in part by the FAK signaling pathway. Indeed, it has been reported that resveratrol is apparently rapidly metabolized in vivo [62]. Although further investigations are needed to understand more on the signaling mechanism of resveratrol, our findings suggest that a molecular signaling pathway relationship between resveratrol-induced Sirt1 and FAK activation, which might be a novel therapeutic target for crucial regulation of cell proliferation, migration and metastases in colon cancer.
